# The prognosis of hepatocellular carcinoma after curative hepatectomy in young patients

**DOI:** 10.18632/oncotarget.4330

**Published:** 2015-06-02

**Authors:** Sang Yun Ha, Insuk Sohn, Soo Hyun Hwang, Jung Wook Yang, Cheol-Keun Park

**Affiliations:** ^1^ Department of Pathology and Translational Genomics, Samsung Medical Center, Sungkyunkwan University School of Medicine, Seoul, Korea; ^2^ Biostatistics and Clinical Epidemiology Center, Research Institute for Future Medicine, Samsung Medical Center, Seoul, Korea

**Keywords:** oncology, carcinogenesis, age, cell cycle, liver

## Abstract

Age at diagnosis is a reported prognostic factor in a variety of solid cancers. In hepatocellular carcinomas (HCCs), several previous studies focused on patient age, but demonstrated inconclusive results on prognosis of young patients. Clinical outcome may differ according to the balance between tumor's own biologic behavior and underlying liver function thus explaining the inconclusive results in previous studies. In this study, we enrolled 282 patients who underwent curative hepatectomy for primary HCCs and had Child Pugh Class A, representing good liver function. Clinicopathologic features were compared between patients aged ≤40 years (young age group) and those aged >40 years (old age group). Thirty-five patients (12.4%) were classified as the young age group and showed larger tumor size (>5cm), higher Edmondson grade, more frequent intrahepatic metastasis and higher alpha-fetoprotein level (>200ng/mL) than old age group. Young age group showed shorter disease specific survival than the old age group. Symptomatic presentation without surveillance was more frequent in the young age group than old age group (45.7% *vs*. 23.9%). In gene expression profiling analysis, 69 differentially expressed genes between young and old age groups were generated and these genes were mostly associated with cell cycle or cell division. Mitotic rate was significantly higher in HCCs of young patients than those of old patients. In conclusion, HCCs in young patients have distinct clinicopathologic features. Poor prognosis in the young age group could be explained by late detection as well as their own aggressive tumor biology.

## INTRODUCTION

Hepatocellular carcinoma (HCC) is one of the most common solid cancers and the second leading cause of cancer-related deaths globally, with only 7% of 5 year survival rate [1]. The majority of HCC occurs in patients with underlying chronic liver disease [2] and surveillance for HCC detection in these risk groups is an important issue, especially the starting age of surveillance. Although, HCC usually occurs in middle aged and elderly patients, the peak age of incidence is different in various countries [3]. For example, the age at diagnosis in HCC patients is lower in hepatitis B-endemic country, such as Korea, than in hepatitis C-endemic areas, such as Western countries [3].

Age at diagnosis is a reported prognostic factor in a variety of solid cancers. Young patients with gastric or breast cancer have more aggressive disease and poorer prognosis than older ones [4, 5]. Conversely, young patients have a better clinical outcome than their elderly counterpart in thyroid papillary carcinoma and colorectal cancer [6, 7]. In HCC, the prognosis of young patients is controversial. Some authors reported better survival rates in young HCC patients, as compared to older patients [3, 8-10], while other studies showed opposite or arbitrary results [11-15].

Nevertheless, common clinicopathologic findings of young HCC patients have been reported in several studies, despite differences in clinical outcome. Young patients have more frequent HBV infection, less frequent HCV infection and higher alpha-fetoprotein (AFP) levels [3, 8-15]. They have relatively larger size and more advanced stage tumor than their elderly counterparts, while background liver function in young patients is relatively well preserved, as compared to older patients [3, 8, 10-14]. Clinical outcome may differ according to the balance between tumor's own biologic behavior and liver function thus explaining the inconclusive results in previous studies.

We evaluated the prognosis of young patients among the 282 HCC patients with long term follow-up. We controlled for liver function by enrolling patients who underwent curative hepatectomy for primary HCCs and had Child Pugh Class A representing good liver function. Therefore, we determined the net prognostic effect of HCC in young patients.

## RESULTS

Thirty-five of the 282 patients (12.4%) belonged to the young patient group. The comparison of clinicopathologic parameters between young and old age groups was summarized in Table 1. The frequency of young patients was higher in female than in male (34.3% *vs*. 14.6%, *p* = 0.004). Tumor sized > 5cm was more frequently found in the young than old patient group (54.3% *vs*. 35.2%, *p* = 0.029). The young patient group had more frequent high Edmondson grade (*p* < 0.001), intrahepatic metastasis (*p* = 0.029) and elevated serum AFP (*p* = 0.048) than the old age group. Young patient group showed tendencies of higher HBV infection rate (88.6% *vs*. 74.9%) and lower HCV infection rate (0% *vs*. 9.7%) than the old patient group. The frequencies of background cirrhosis were similar between the 2 age groups (48.6% *vs*. 50.6%).

**Table 1:** The association between patient group by age and clinicopathologic parameters

			Age		
		Total	≤40 years	> 40 years	*p* value
**Gender**					
	Female	48 (17.0)	12 (34.3)	36 (14.6)	0.004
	Male	234 (83.0)	23 (65.7)	211 (85.4)	
**Tumor size**					
	≤ 5cm	176 (62.4)	16 (45.7)	160 (64.8)	0.029
	> 5cm	106 (37.6)	19 (54.3)	87 (35.2)	
**Edmondson grade**					
	I	32 (11.3)	1 (2.9)	31 (12.6)	<0.001*
	II	226 (80.1)	25 (71.4)	201 (81.4)	
	III	24 (8.5)	9 (25.7)	15 (6.1)	
**Microvasular invasion**					
	(−)	130 (46.1)	12 (34.3)	118 (47.8)	0.134
	(+)	152 (53.9)	23 (65.7)	129 (52.2)	
**Major portal vein invasion**					
	(−)	271 (96.1)	32 (91.4)	239 (96.8)	0.144*
	(+)	11 (3.9)	3 (8.6)	8 (3.2)	
**Intrahepatic metastasis**					
	(−)	218 (77.3)	22 (62.9)	196 (79.4)	0.029
	(+)	64 (22.7)	13 (37.1)	51 (20.6)	
**Multicenteric occurrence**					
	(−)	265 (94.0)	34 (97.1)	231 (93.5)	0.704*
	(+)	17 (6.0)	1 (2.9)	16 (6.5)	
**AJCC T stage**					
	1	122 (43.3)	12 (34.3)	110 (44.5)	0.365*
	2	112 (39.7)	14 (40.0)	98 (39.7)	
	3	42 (14.9)	8 (22.9)	34 (13.8)	
	4	6 (2.1)	1 (2.9)	5 (2.0)	
**BCLC stage**					
	0-A	162 (57.4)	14 (40.0)	148 (59.9)	0.051*
	B	107 (37.9)	18 (51.4)	89 (36.0)	
	C	13 (4.6)	3 (8.6)	10 (4.0)	
**Albumin level, g/dL**					
	>3.5	21 (7.4)	4 (11.4)	17 (6.9)	0.310*
	≤ 3	261 (92.6)	31 (88.6)	230 (93.1)	
**AFP level, ng/mL**					
	≤200	172 (61.0)	16 (45.7)	156 (63.2)	0.048
	>200	110 (39.0)	19 (54.3)	91 (36.8)	
**Etiology**					
	Non-viral	38 (13.5)	4 (11.4)	34 (13.8)	0.203*
	HBV	216 (76.6)	31 (88.6)	185 (74.9)	
	HCV	24 (8.5)	0 (0)	24 (9.7)	
	HBV and HCV	4 (1.4)	0 (0)	4 (1.6)	
**Liver cirrhosis**					
	(−)	140 (49.6)	18 (51.4)	122 (49.4)	0.822
	(+)	142 (50.4)	17 (48.6)	125 (50.6)	

* By Fisher's exact test, otherwise by chi square test

**Figure 1:**
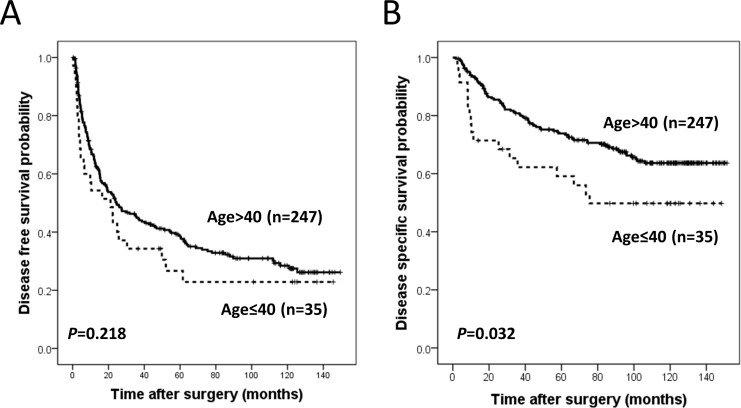
Kaplan Meier survival curves for disease free survival **A.** and disease specific survival **B.** in patient groups according to age.

Young patient group showed shorter disease specific survival (DSS) (*p* = 0.032) and a tendency of shorter disease free survival (DFS) (*p* = 0.218), as compared to the old patient group (Figure 1). On multivariate analysis including covariables (mode of presentation, tumor size, Edmondson grade, microvascular invasion, major portal vein invasion, intrahepatic metastasis, serum albumin level, serum AFP level and etiology) with statistical significance in univariate analysis, patient age failed to demonstrate the statistical significance for both DFS and DSS (Table 2).

**Table 2:** Multivariate analysis for recurrence free survival and disease-specific survival

		Disease Free Survival			Disease Specific Survival		
		HR	95% CI	*p* value	HR	95% CI	*p* value
**Age**	≤40 years *vs* >40 years	0.996	0.631-1.571	0.85	1.093	0.615-1.943	0.761
**Presentation**	Symptomatic *vs* others	1.166	0.816-1.667	0.399	1.35	0.851-2.141	0.203
**Tumor size**	>5cm *vs* ≤ 5cm	0.949	0.665-1.353	0.771	1.398	0.857-2.279	0.18
**Edmondson grade**	III *vs* I+II	1.403	0.830-2.372	0.206	1.336	0.712-2.509	0.367
**Microvasular invasion**	yes *vs* no	1.335	0.921-1.933	0.127	1.5	0.84602.662	0.165
**Major portal vein invasion**	yes *vs* no	0.722	0.350-1.490	0.379	1.15	0.534-2.476	0.721
**Intrahepatic metastasis**	yes *vs* no	3.693	2.426-5.623	<0.001	3.444	2.041-5.809	<0.001
**Albumin level, g/dL**	≤3.5 *vs* >3.5	2.054	1.157-3.645	0.014	2.714	1.413-5.210	0.003
**AFP level, ng/mL**	>200 *vs* ≤200	1.338	0.97601.834	0.07	1.157	0.744-1.800	0.516
**Etiology^1^**	Viral *vs* non-viral	1.655	0.996-2.750	0.052			

HR, Hazard Ratio; CI, Confidence Interval

1 Etiology was not a significant factor in univariate analysis for disease specific survival and was not included in multivariate analysis.

### Factors for shorter disease-specific survival in young *vs*. old patients

We formulated 2 hypotheses for shorter DSS in young patients: 1. Late tumor detection at an advanced stage with conspicuous symptoms results from less frequent surveillance of HCC in young *vs*. old patients; 2. HCCs in young patients have their own more aggressive behavior than those in old patients.

Therefore, we investigated the mode of presentation such as symptomatic, surveillance, or incidental. Young patients showed more frequent symptomatic presentation and less frequent presentation by surveillance than old patients (Table 3).

**Table:** Association between presentation mode and age group

Presentation mode		Age		
	Total	≤40 years	> 40 years	*p* value
**Symptomatic**	75 (26.6)	16 (45.7)	59 (23.9)	0.021
**Surveillance**	140 (49.6)	14 (40.0)	126 (51.0)	
**Incidental**	67 (23.8)	5 (14.3)	62 (25.1)	

**Figure 2:**
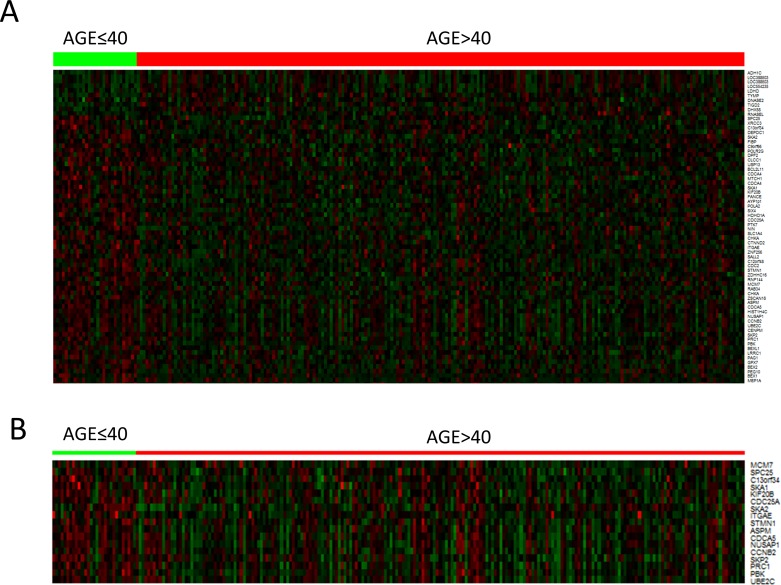
Heatmap of differentially expressed genes in hepatocellular carcinoma of young and old patient groups **A.** 69 differentially expressed genes (*q* value < 0.2) **B.** 18 cell cycle related genes.

Next, we compared gene expression profiles in young and old patient groups after adjusting for significant confounding factor such as Edmondson grade, etiology, cirrhosis, AJCC T stage, albumin level and AFP level. As a result, 69 DEGs were generated (*p* < 0.001, *q* < 0.2) (Figure 2A and Supplementary Table 1) and these DEGs were mostly involved in pathways associated with cell cycle or cell division (Figure 2B and Table 4). Mitotic rate was also significantly higher in HCCs of young patients than those of old patients (*p* = 0.032).

**Table 4:** Functional annotation chart of 69 differentially expressed genes between young and old patient group

Category	Term	Count	%	*P* value	FDR
GOTERM_BP_FAT	GO:0000278~mitotic cell cycle	15	25.0	5.30E-11	7.55E-08
GOTERM_BP_FAT	GO:0000279~M phase	14	23.3	1.70E-10	2.43E-07
SP_PIR_KEYWORDS	mitosis	11	18.3	1.99E-10	2.31E-07
GOTERM_BP_FAT	GO:0022403~cell cycle phase	15	25.0	2.34E-10	3.33E-07
SP_PIR_KEYWORDS	cell division	12	20.0	4.13E-10	4.79E-07
GOTERM_BP_FAT	GO:0000280~nuclear division	12	20.0	4.41E-10	6.29E-07
GOTERM_BP_FAT	GO:0007067~mitosis	12	20.0	4.41E-10	6.29E-07
GOTERM_BP_FAT	GO:0000087~M phase of mitotic cell cycle	12	20.0	5.34E-10	7.62E-07
GOTERM_BP_FAT	GO:0048285~organelle fission	12	20.0	6.76E-10	9.63E-07
GOTERM_BP_FAT	GO:0051301~cell division	12	20.0	9.66E-09	1.38E-05
SP_PIR_KEYWORDS	cell cycle	13	21.7	1.26E-08	1.45E-05
GOTERM_BP_FAT	GO:0007049~cell cycle	17	28.3	1.27E-08	1.82E-05
GOTERM_BP_FAT	GO:0022402~cell cycle process	15	25.0	1.30E-08	1.85E-05
GOTERM_CC_FAT	GO:0044427~chromosomal part	9	15.0	1.42E-05	0.0158605
GOTERM_CC_FAT	GO:0005694~chromosome	9	15.0	5.00E-05	0.0557695
SP_PIR_KEYWORDS	nucleus	28	46.7	5.31E-05	0.0614677
GOTERM_CC_FAT	GO:0005819~spindle	6	10.0	6.95E-05	0.0775141
SP_PIR_KEYWORDS	microtubule	7	11.7	7.44E-05	0.0861766
GOTERM_CC_FAT	GO:0005876~spindle microtubule	4	6.7	8.40E-05	0.0936886

## DISCUSSION

The results of the several reports on the patient age in HCCs were summarized in Table 5. The prognosis of young HCC patients were inconclusive in previous studies [3, 8-15]. Among the 9 listed studies, better survival was reported in 4 studies, poorer survival in 3, and comparable or paradoxical influence on survival in 2. The criteria of young age was 40 years old in 7 studies, and 30 in 2 studies. The mode of diagnosis and modality for treatment were diverse. Nevertheless, common clinicopathologic findings in young patients have been suggested in many studies. More frequent HBV and less frequent HCV infection in young patients were reported in 7 of 9 studies, and higher AFP levels in 6 studies. Advanced tumor stage was found in 7 studies, while relatively good liver function was documented in 5 studies. These distinct clinicopathologic features were likewise observed in our study. The results collectively suggest that HCCs in young patients have their own biologic behavior.

**Table 5:** Summary of previous studies regarding hepatocellular carcinoma in young patients

Author	Year	Country	Total N	Cutoff (years)	Proportion of young patients	Diagnosis	Treatment	Clinicopathologic findings	Prognostic finding
Shimada et al. [14]	2013	Japan	811	40	31 (3.8%)	Pathologically	liver resection	more frequent HBV, less frequent HCV, higher AFP levels, more cases with a maximum tumor size of >=5cm, more microscopic tumor thrombus in portal vein, more intrahepatic metastasis	not significant in OS and
Niederle et al. [9]	2012	Germany	1108	40	25 (2%)	Pathologically or clinically	variable	less common underlying chronic liver disease in young age group, higher AFP levels, more frequent fibrolamellar carcinoma	better OS (*p* = 0.048)
Takeishi et al. [10]	2011	Japan	610	40	13 (2.1%)	Pathologically	curative resection	more frequent HBV, less frequent HCV, higher platelet count, higher AFP levels, larger size, poorly differentiated, more portal vein invasion, more advanced stage, shorter operative time	tendency to better OS (*p* = 0.057) not significant in DFS (*p* = 0.762)
Chang et al. [8]	2008	Singapore	638	40	55 (8.6%)	Pathologically or clinically	variable	more frequent HBV, less frequent HCV, higher AFP levels, higher albumin, less cirrhosis, better child-Pugh class, more portal vein invasion, more advanced stage	tendency to better OS (*p* = NS) better OS in stage I-III (*p* = 0.025)
Yamazaki et al. [15]	2007	Japan	NA	40	20	Pathologically or clinically	variable	HBV 75%, Child Pugh Grade A 85%	eleven patients out of 20 died within 1 year
Cho et al. [12]	2007	Korea	320	30	71 (22%)	NA	NA	more frequent HBV, less frequent HCV, higher AFP levels, less cirrhosis, more advanced stage, more symptomatic patients	poor survival than other age groups (*p* = 0.007) - not significant after stage adjustment
Chen et al. [11]	2006	Taiwan	11,312	40	1229 (10.9%)	Pathologically or clinically	variable	more frequent HBV, less frequent HCV, larger size	paradoxical influence on survival worse 1 year survival (*p* < 0.001) better survival after 1 year (*p* < 0.001)
Kim et al. [3]	2006	Korea	4,234	30	38 (0.9%)	Pathologically or clinically	variable	low frequency of smoking history, more frequent HBV, less frequent HCV, higher AFP levels, well-preserved liver function, larger tumor size, more advanced stage, more frequent application of surgical resection and chemotherapy as initial treatment.	better OS than age group (40-59) (*p* = 0.04) similar OS with age group (>=60) better OS than other age groups in TNM stage I and II (*p* = 0.04)
Lam et al. [13]	2004	HongKong	1863	40	121 (6.5%)	clinically	variable	more frequently presented with pain, hepatomegaly, ruptured HCC/less frequently detected by routine screening/better Child-Pugh grading and ICG test/higher AFP level, larger tumor size, more frequent metastasis	shorter OS (*p* = 0.004)

The inconclusive effect on prognosis may be explained by the balance between aggressive tumor factor and good underlying liver function. We enrolled patients who were classified as a Child Pugh class A who underwent curative resection for primary HCCs. Therefore, we were able to control the effect of underlying liver function on patient survival. As a result, HCCs in young patients were associated with larger tumor size, higher Edmondson grade, more frequent intrahepatic metastasis and higher AFP level. These results were consistent with previous studies. The young age group showed shorter DSS than the old age group (*p* = 0.032), probably due to pure tumor effect with offsetting underlying liver function.

The causes of the aggressive phenotype of HCCs in young patients are not clear. We confirmed that symptomatic presentation without surveillance was more frequent in the young *vs*. old age group possibly due to late detection after progression to the advanced stage. This phenomenon was also documented in 2 previous studies by Cho et al. and Lam et al. [12, 13]. We identified the biologic difference of HCCs between young and old age groups, and compared gene expression profiles between the 2 groups after controlling for other confounding factors. As a result, DEGs were mostly associated with cell cycle or cell division. Further validation showed that the mitotic index was higher in the young patient group, as compared to old patient group. These results indicated that HCCs in young patients have their own aggressive behavior associated with increased cell division. A similar phenomenon was reported in a previous study by Geigl et al. on gene expression pattern according to aging using fibroblast cell lines and lymphocytes cultures from young and old patients [16]. They reported that a number of genes differentially expressed in aged cells were involved in both cell cycle and proliferation.

In fact, age was not an independent predictor of DFS and DSS in the multivariate analysis of this study, and poor outcome in young patients could be explained by advanced staging at the time of diagnosis. However, young patients showed shorter DSS than old patients in BCLC stage B-C group (Supplementary Figure 1B, *p* = 0.012) and a tendency of shorter DFS than old patients in BCLC 0-A group (Supplementary Figure 1C). These results suggest that poor outcome in young patients could not be fully originated from advanced staging of HCC at the time of diagnosis, supporting the probable role of their own biologic features such as increased cell division.

American Association for the Study of Liver Diseases (AASLD) recommend HCC surveillance for all cirrhotic HBV patients, Asian male HBV patients older than 40 years and Asian female HBV patients elder than 50 years [17]. In Korea, HCC surveillance is recommended in patients older than 40 years with cirrhosis or chronic HBV or HCV hepatitis by the Ministry of Health and Welfare and National Cancer Center. In this study, young HCC patients might have been subject to advanced stage on initial diagnosis because of less likelihood of being under surveillance, which led to late detection, and poor prognosis. For this reason, HCC surveillance begins at much younger ages in high risk group of patients in Korea. Whether this approach is cost effective or not remains to be determined and should be further investigated in the future study.

This study is a single center based retrospective study performed in Korea where HBV is the major etiology of HCC. Therefore, it has a limitation in the aspect of generalizability. Further studies are needed to validate major findings in this study.

In conclusion, HCCs in young patients have distinct clinicopathologic features. Poor prognosis in the young age group is possibly due to late detection as well as their own aggressive tumor biology.

## MATERIALS AND METHODS

### Patient population and clinicopathologic information

Initially, a total of 290 curative resected and pathologically confirmed primary HCCs at the Samsung Medical Center, Seoul, Korea from July 2000 to May 2006, were enrolled in the study. Eight cases with preoperative treatment such as trans-arterial chemoembolization, radiofrequency ablation or radiotherapy were excluded, and finally 282 patients were included in the study. Curative resection was defined as complete resection of all tumor nodules with clear microscopic resection margins and no residual tumors as indicated by a computed tomography scan 1 month after surgery.

We reviewed medical records of clinical parameters including age, gender, mode of presentation (surveillance, symptomatic, incidental), history of alcohol intake, and the results of laboratory test including serology for hepatitis virus A, B, C and D, AFP, and serum albumin. Surveillance was defined as a every 6-month screening with abdominal ultrasound and serum AFP for early detection of HCC in high risk patients with cirrhosis, HBsAg (+) or Anti-HCV Ab (+). Histopathologic features of HCCs such as histologic differentiation, microvascular invasion, major portal vein invasion, intrahepatic metastasis, multi-centric occurrence, and non-tumor liver pathology were reviewed by 2 pathologists (SYH and CKP). Histologic grading of HCCs were determined according to the criteria of Edmondson and Steiner [11]. Intrahepatic metastasis and multi-centric occurrence were determined according to the criteria of the Liver Cancer Study Group of Japan [12]. All patients were staged using the American Joint Committee on Cancer (AJCC) staging system [13] and Barcelona Clinic Liver Cancer (BCLC) staging classification [14]. The Institutional Review Board of Samsung Medical Center granted approval for this study.

The patients were followed-up every 3 months after surgery. The tumor recurrence was detected by 3 phase dynamic computed tomography scans or magnetic resonance imaging. The median follow-up period was 119.8 months (range 14.0-151.4 months) for survivors and the follow-up period for recurrence was at least 24 months. DFS was defined from the date of operation until the detection of tumor recurrence. DSS was defined as the interval between the date of surgery and the date of HCC-related death. It was defined as: 1) the tumor occupying > 80% of the liver; 2) portal venous tumor thrombus proximal to the second bifurcation; 3) obstructive jaundice due to the tumor; 4) distant metastases; and 5) variceal hemorrhage with portal venous tumor thrombus proximal to the first bifurcation [18].

### Comparison between patient groups according to patient age

We defined young patients as aged ≤ 40 years based on previous studies [8-11, 13-15]. Clinicopathologic parameters and survival in the young patient group were compared to those in patient group with age > 40 years.

### Microarray data analysis

For comparison of gene expression profiling in young and old age group, we used our previously published microarray data of 240 cases [19]. A total of 240 cases were included in this study cohort. The expression data has been deposited in Gene Expression Omnibus (GSE 36376, http://www.ncbi.nlm.nih.gov/geo/). Multiple linear regression model was applied for adjusting confounding factors as follow: Edmondson grade, etiology, cirrhosis, AJCC T stage, albumin level, and AFP level. False discovery rate was applied for multiple correction. Differentially expressed genes (DEGs) selected by the criteria of *p* < 0.001 and *q* < 0.2, were imported into DAVID (the database for annotation, visualization, and integrated discovery) bioinformatics resources [20] to document biologic meaning associated with these gene lists.

### Evaluation of mitotic rates

As a validation of gene expression profiling results, we compared the mitotic indices in young and old patient groups, using our previously reported data [21]. Multiple linear regression model was applied for adjusting confounding factors, similar to the microarray data analysis.

### Statistical analysis

The association between age groups and clinicopathologic parameters was analyzed using the chi-square test, Fisher's exact test or Cochran Armitage test. Survival analysis was performed using the Kaplan-Meier method, and the difference in survival rates was assessed by the log-rank test. The Cox proportional hazard regression model was used to assess the association between clinicopathologic factors and survival time. Significant prognostic factors identified by univariate analysis were entered into multivariate analysis. Proportional hazard assumption was checked by graphical method. We confirmed that variables in Cox proportional hazard model were constants that do not depend on time. All statistical analyses were performed using SPSS software (SPSS Inc., Chicago, IL, USA) or R software (version 3.03). *P*-values < 0.05 were considered to be statistically significant.

## SUPPLEMENTARY FIGURES AND TABLE


