# Supplementary material for: The prognosis of hepatocellular carcinoma after curative hepatectomy in young patients
Source: Oncotarget. 2015 Jun 2;6(21):18664–73. doi: 10.18632/oncotarget.4330 (PMC4621918; doi:10.18632/oncotarget.4330)
Supplement: Supplementary file 1 [file oncotarget-06-18664-s001.pdf]

The prognosis of hepatocellular carcinoma after curative hepatectomy in young patients

Supplementary Material

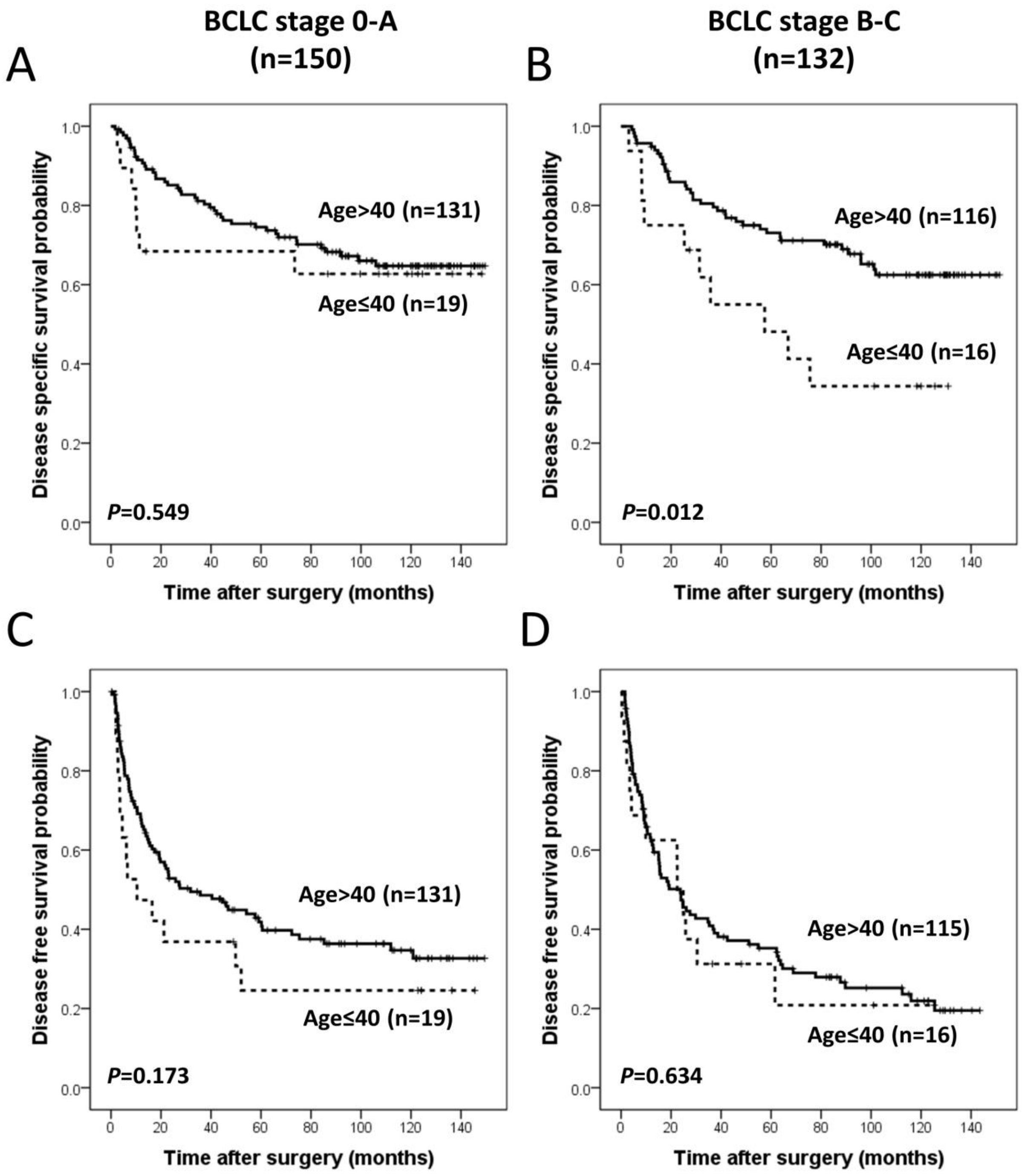

Figure S1: Kaplan Meier survival curves in patient groups according to age; **A.** disease specific survival in BCLC stage 0-A; **B.** disease specific survival in BCLC stage B-C; **C.** Disease free survival in BCLC stage 0-A; **D.** Disease free survival in BCLC stage B-C
